# Supplementary material for: Rare Occurrence of Blastocystis in Pet Animals and Their Owners in the Pomeranian Voivodeship in Poland in the Light of Literature Data
Source: J Clin Med. 2022 May 25;11(11):2975. doi: 10.3390/jcm11112975 (PMC9181306; doi:10.3390/jcm11112975)
Supplement: Supplementary file 1 [file jcm-11-02975-s001.zip › jcm-1714936-supplementary.pdf]

Table S1. Summary of data on *Blastocystis* in dogs, calculated on the basis of the data from Table 1.

Dogs	Positive Samples	Negative Samples	Percentage of Positive Samples
Kept indoors	120	1508	7,37%
From rural areas	2	207	0,96%
Shelter-resident	117	306	27,66%
Stray	56	274	20,44%
